# Challenges and Solutions in Delivering Preventive Periodontal Care to Older Adults in Community and Long‐Term Residential Care Settings: A Scoping Review

**DOI:** 10.1155/ijod/8633133

**Published:** 2026-09-29

**Authors:** Ankita Chakraborty, Deepa Kamath G, Nina Shenoy, Anwesha Basu, Aradhya Sinha

**Affiliations:** ^1^ Department of Periodontology, Manipal College of Dental Sciences Mangalore, Manipal Academy of Higher Education, Manipal, India, manipal.edu; ^2^ Department of Periodontics, Nitte (Deemed to be University), AB Shetty Memorial Institute of Dental Sciences (ABSMIDS), Mangaluru, Karnataka, 575018, India, nitte.edu.in

**Keywords:** challenges, community settings, older adults, oral health promotion, oral hygiene, periodontal care, periodontal diseases, preventive care, scoping review, solutions

## Abstract

**Background:**

Preventive periodontal care is essential for older people in the community to reduce the risk of tooth loss, systemic diseases, and decreased quality of life. Despite its importance, older people in assisted living, home‐based care (HBC), daycare, and other community settings face barriers to effective preventive periodontal care, including mobility, cognitive, and caregiver knowledge issues. Although these challenges are being recognized more and more, the evidence on barriers and solutions for delivering consistent preventive periodontal care remains scattered across different study designs and care contexts.

**Aim and Objective:**

The aim of this scoping review is to map the existing literature and systematically identify the key challenges at patient, caregiver, and institutional levels, along with evidence‐based solutions—including behavior change techniques (BCTs), tools, and interventions—for delivering preventive periodontal care to older adults in community and mixed residential settings.

**Methods:**

The Joanna Briggs Institute scoping review method, along with the PRISMA‐ScR, was followed to identify the key issues, solutions, and findings related to the implementation of preventive periodontal care in older people in the community. The inclusion criteria were older people aged 60 years or more, interventions, community, or mixed settings, and the languages were restricted to English. The search was conducted for articles from 2010 or later. Two‐phase selection was used, which yielded 32 articles. This was followed by data extraction, including study design, settings, population, interventions, outcomes, issues, solutions, thematic synthesis, etc.

**Results:**

Thirty‐two studies were included (12 cross‐sectional, 8 RCTs/quasi‐RCTs, 6 systematic reviews/protocols, and 6 others). Included studies were conducted mainly in nursing homes (*n* = 15), with 10 in community and 7 in mixed settings. Key challenges were identified at the resident, caregiver, and institutional/systemic levels. Resident‐level challenges included cognitive and functional limitations, while caregiver‐level barriers included gaps in knowledge and the relatively low priority given to oral care. At the institutional and systemic levels, workload, limited access to professional dental care, and the lack of standardized oral‐care protocols were frequently reported. Solutions included carer education with BCTs, standardized tools (e.g., Oral Health Assessment Tool [OHAT]), visual aids, AI‐supported screening, regular professional outreach brushing, and interprofessional models, showing modest short‐term improvements in plaque index (PI), gingival index (GI), and bleeding on probing (BOP).

**Conclusions:**

Persistent challenges underscore the need for integrated, accessible strategies to enhance preventive care. Future research should prioritize cost‐effectiveness, community‐focused RCTs, and long‐term evaluations to improve oral health outcomes in aging populations.

## 1. Introduction

The global population is currently undergoing a significant demographic shift, whereby the number of people aged 60 years or older is expected to double in the next 30 years, accounting for 22% of the total population by 2050 [1, 2]. This is a major challenge to the existing healthcare infrastructure, especially regarding the issue of oral health, which, unlike other medical fields, has traditionally followed the reactive rather than preventative model [3]. Periodontal disease (PD) and dental caries are now recognized as the most prevalent noncommunicable diseases, surpassing the combined prevalence of mental diseases, cardiovascular diseases, diabetes, respiratory diseases, and cancer [1, 3]. Even among relatively healthy individuals, inadequate oral hygiene has been reported. In a study conducted in Bern, Switzerland, a substantial proportion of participants had an Approximal Plaque Index (API) above 50%, despite most participants reporting that they brushed their teeth twice daily [4, 5]. This finding highlights that reported toothbrushing frequency alone may not necessarily reflect adequate plaque control.

Preventive periodontal care refers to a set of strategies and interventions aimed at preventing the onset and progression of gingival and PDs through the control of bacterial plaque biofilm. It comprises daily oral hygiene practices such as toothbrushing, interdental cleaning, tongue cleaning, and the use of adjunctive chemical agents like chlorhexidine mouth rinses. In addition, it includes regular professional periodontal assessments using clinical indicators such as the plaque index (PI), gingival index (GI), bleeding on probing (BOP), and probing pocket depth (PPD), along with supportive professional interventions such as scaling and root surface debridement when indicated.

Preventive periodontal care also involves patient and/or caregiver education regarding the significance of oral hygiene maintenance, behavior change techniques (BCTs) to support daily oral hygiene maintenance, and the establishment of long‐term maintenance protocols to sustain periodontal health.

New evidence is increasingly coming to light regarding the complex relationship between PD and systemic health [6, 7]. Evidence suggests that PDs are associated with several systemic conditions through shared inflammatory pathways and biological mechanisms; however, many of these relationships remain associative rather than definitively causal. Consequently, maintaining periodontal health is increasingly recognized as an important component of healthy aging and comprehensive healthcare.

Click or tap here to enter text. In this scoping review, community settings refer to environments where older adults live independently or semi‐independently in their own homes or with home‐based support services, including home care recipients and community‐dwelling individuals [8–10].

Mixed residential settings comprise a range of formal care environments such as assisted living facilities, nursing homes, and residential senior care facilities, where older adults with varying levels of dependency receive accommodation and daily assistance [2–5]. These include both assisted living and more intensive long‐term care (LTC) homes, where preventive periodontal care is often delivered through caregivers and institutional protocols [7, 11, 12].

As the older adult transitions from independent to care‐dependent, the first functional skill affected is oral hygiene maintenance [4]. Cognitive impairments, as measured by decreased SMMSE scores, are positively correlated with decreased numbers of natural teeth and reduced masticatory ability [4]. This sets off the downward spiral of memory loss, loss of motor control, and apraxia, leading to oral hygiene neglect, which in turn accelerates cognitive impairments and nutritional deficiencies [4, 13]. Oral hygiene is often deprioritized in the community and LTC settings and is often classified as “missed nursing care.” Care providers often view oral hygiene as merely grooming and not an essential infection control measure [4, 14].

Older adults in the community setting, such as home‐based care (HBC) and assisted living, experience unique barriers to oral health care [5, 8]. Studies from Germany indicate that HBC recipients may experience even lower oral health outcomes than their nursing home counterparts due to the lack of professional oversight and dental contracts in HBC settings [9].

Older adults face several barriers to maintaining adequate oral hygiene across different levels of care. At the patient level, high rates of care‐resistant behaviors, such as biting, screaming, and refusal of oral care, have been reported among older adults with dementia, making routine oral hygiene difficult [7]. At the provider level, knowledge gaps among medical practitioners and caregivers, along with cultural beliefs that tooth loss is an inevitable part of aging, may contribute to inadequate oral care [2, 8]. At the institutional level, high staff turnover and the lack of standardized tools and protocols for monitoring routine oral care have also been identified as important gaps [4, 11].

Current periodontal research literature underscores the significance of proper preventive care in the maintenance of periodontal health. Current evidence shows that regular supportive periodontal care and successful control of periodontal inflammation help ensure periodontal stability, while the effectiveness of particular therapy methods can depend on the clinical state and therapy chosen [15]. It has also been found that the effectiveness of adjunctive antimicrobial therapies in the case of periodontal and peri‐implant diseases is not constant [16, 17].

Thus, recent literature has highlighted the importance of individualized supportive periodontal care, evidence‐based maintenance strategies, and preventive interventions for preserving periodontal health over time. Nevertheless, implementing these preventive approaches among older adults in community and residential care settings remains challenging because of patient‐, caregiver‐, institutional‐, and system‐level barriers. It is especially significant in the case of elderly people whose ability to practice regular oral hygiene, use the services of professionals, and maintain preventive care depends on their physical capabilities, degree of dependency, availability of caregivers, and other peculiarities of care settings. Thus, it is important not only to understand the clinical approach to periodontal prevention but also the barriers and strategies for its implementation.

Aim of the study: The aim of this scoping review is to map the existing literature and systematically identify the key challenges at patient, caregiver, and institutional levels, along with evidence‐based solutions—including BCTs, tools, and interventions—for delivering preventive periodontal care to older adults in community and mixed residential settings.

## 2. Materials and Methods

This study is a scoping review designed to compile and chart the available research evidence, outlining the scope, features, and extent of studies on the topic, and was conducted in accordance with the Joanna Briggs Institute guidelines [18]. This review also aligns with PRISMA‐ScR standards “Preferred Reporting Items for Systematic Reviews and Meta‐Analyses Extension for Scoping Reviews” [18].

The review is guided by this research question framed in the PCC structure: “What are the primary patient, provider, and institutional challenges in delivering preventive periodontal care to older adults in community and residential settings, and what evidence‐based solutions exist to overcome these barriers?”

The review protocol was retrospectively registered publicly on the Open Science Framework (OSF) on March 12, 2026, following completion of the database searches conducted on January 5, 2026.

It is accessible via https://doi.org/10.17605/OSF.IO/Q7H4C (DOI: 10.17605/OSF.IO/Q7H4C).

Initial database searches in PubMed, Scopus, EBSCO, and protocol registries confirmed no prior or ongoing reviews addressing this specific question.

### 2.1. Search Strategy

Electronic searches were conducted on January 5, 2026, in PubMed, Scopus, and the EBSCOhost platform MEDLINE via EBSCO for studies published between January 2010 and January 2026. The restriction to publications from 2010 onwards was applied to capture contemporary models of preventive periodontal care relevant to current community and residential aged care services. Only studies published in English were considered. Search terms: ("preventive periodontal care” OR “oral hygiene” OR “periodontal prevention") AND ("older adults” OR “elderly” OR “geriatric") AND ("community settings” OR “assisted living” OR “home‐based” OR “homebound") AND ("challenges” OR “barriers” OR “solutions” OR “interventions").

A search strategy was developed for each database using relevant keywords and MeSH terms, where applicable. The search strategy was adapted for each database. The complete search strategy for each database is presented in Table 1. The retrieved records were exported into Rayyan (https://www.rayyan.ai/) for duplicate removal and screening.

**Table 1:** Search strategy for databases used in the scoping review on preventive periodontal care for older adults in community settings.

Search engine	Search string	Date
PubMed	(“Periodontal Diseases”[Mesh] OR periodontal[tiab] OR periodontitis[tiab] OR “periodontal care”[tiab] OR “oral hygiene”[tiab]) AND (“Preventive Health Services”[Mesh] OR preventive [tiab] OR prevention[tiab] OR “preventive care”[tiab] OR “oral health promotion”[tiab]) AND (“Aged”[Mesh] OR “Aged, 80 and over”[Mesh] OR older adults[tiab] OR elderly[tiab] OR seniors[tiab] OR “older people”[tiab]) AND (“Community Health Services”[Mesh] OR “Residential Facilities”[Mesh] OR “Nursing Homes”[Mesh] OR “long‐term care”[tiab] OR “nursing home ^∗^”[tiab] OR “residential aged care”[tiab] OR “community setting ^∗^”[tiab] OR “community‐dwelling”[tiab]) AND (“Caregivers”[Mesh] OR nurses[tiab] OR “nursing staff”[tiab] OR “care staff”[tiab] OR “direct care staff”[tiab] OR caregiver ^∗^[tiab]) AND (challenge ^∗^[tiab] OR barrier ^∗^[tiab] OR obstacle ^∗^[tiab] OR facilitators[tiab] OR implementation[tiab] OR solution ^∗^[tiab] OR “behavior change”[tiab] OR “behavior change”[tiab])	January 5th, 2026
Scopus	TITLE‐ABS‐KEY(periodontal OR periodontitis OR “periodontal care” OR “oral hygiene") AND TITLE‐ABS‐KEY(preventive OR prevention OR “preventive care” OR “oral health promotion") AND TITLE‐ABS‐KEY(“older adult ^∗^” OR elderly OR senior ^∗^ OR “older people” OR geriatric ^∗^) AND TITLE‐ABS‐KEY(“community setting ^∗^” OR “community‐dwelling” OR “community health” OR “primary care” OR “nursing home ^∗^” OR “long‐term care” OR “residential aged care” OR “care home ^∗^") AND TITLE‐ABS‐KEY(caregiver ^∗^ OR “nursing staff” OR “care staff” OR “direct care staff” OR nurse ^∗^ OR “allied health") AND TITLE‐ABS‐KEY(challenge ^∗^ OR barrier ^∗^ OR obstacle ^∗^ OR implementation OR “care resistant” OR resistance OR solution ^∗^ OR “behavior change” OR “behavior change” OR “practice change")	January 5th, 2026
MEDLINE (via EBSCOhost)	(MH “Periodontal Diseases+” OR TI(periodontal OR periodontitis OR “periodontal care") OR AB(periodontal OR periodontitis OR “periodontal care") OR TI(“oral hygiene") OR AB(“oral hygiene")) AND (TI(preventive OR prevention OR “preventive care” OR “oral health promotion") OR AB(preventive OR prevention OR “preventive care” OR “oral health promotion")) AND (MH “Aged+” OR TI(“older adult ^∗^” OR elderly OR senior ^∗^ OR “older people” OR geriatric ^∗^) OR AB(“older adult ^∗^” OR elderly OR senior ^∗^ OR “older people” OR geriatric ^∗^)) AND (MH “Community Health Services+” OR MH “Nursing Homes+” OR MH “Long‐Term Care” OR TI(“community setting ^∗^” OR “community‐dwelling” OR “primary care” OR “nursing home ^∗^” OR “residential aged care” OR “care home ^∗^” OR “long‐term care") OR AB(“community setting ^∗^” OR “community‐dwelling” OR “primary care” OR “nursing home ^∗^” OR “residential aged care” OR “care home ^∗^” OR “long‐term care")) AND (MH “Caregivers+” OR MH “Nursing Staff+” OR TI(caregiver ^∗^ OR “care staff” OR “nursing staff” OR “direct care staff” OR nurse ^∗^) OR AB(caregiver ^∗^ OR “care staff” OR “nursing staff” OR “direct care staff” OR nurse ^∗^)) AND (TI(challenge ^∗^ OR barrier ^∗^ OR obstacle ^∗^ OR implementation OR solution ^∗^ OR “care resistant” OR resistance OR “behavior change” OR “behavior change") OR AB(challenge ^∗^ OR barrier ^∗^ OR obstacle ^∗^ OR implementation OR solution ^∗^ OR “care resistant” OR resistance OR “behavior change” OR “behavior change"))	January 5th, 2026

*Note:* Search conducted across PubMed (*n* = 140), Scopus (*n* = 711), and EBSCO (*n* = 47). Column 1—search engine used. Column 2— search strategy. Column 3—date on which search string was generated.

The reference lists of all included studies were also manually screened to identify additional eligible articles. No formal gray literature search was undertaken.

### 2.2. Selection Process

Two independent reviewers (Ankita Chakraborty and Deepa Kamath G) conducted title/abstract screening was conducted against predefined eligibility criteria using the PCC framework (population, concept, context).

Inclusion criteria:

• Population: older adults aged ≥60 years, including frail, dependent, or cognitively impaired individuals.
• Concept: preventive periodontal care, including oral hygiene interventions (toothbrushing, tongue cleaning, chlorhexidine, etc.), periodontal assessments (e.g., PI, GI, BOP, PPD), and related outcomes.
• Context: community settings (homes with or without home care support, assisted living, and daycare centers) or mixed residential settings that include a substantial community component (assisted living facilities, nursing homes, and residential senior care facilities).
• Study types: systematic reviews, RCTs, cross‐sectional, pilot, qualitative studies, and narrative reviews. Primary quantitative studies (randomized controlled trials, quasi‐experimental studies, cross‐sectional and observational studies), qualitative studies, pilot or feasibility studies, systematic reviews, narrative reviews, and study protocols were eligible for inclusion. Qualitative studies were included to identify barriers and implementation issues. Systematic and narrative reviews were included to map the existing evidence base, while study protocols were included to identify ongoing or planned research. These secondary evidence sources were not used as evidence of intervention effectiveness during data synthesis.
• English‐language articles were published from 2010 to January 2026 addressing challenges and/or solutions.

Exclusion criteria:

• Purely institutional/hospital settings without community elements;
• Nonpreventive (restorative/surgical/drug therapy);
• The nonolder adult population (adults aged <60 years);
• Animal studies, editorials, and abstracts without full data;
• Non‐English publication.

In total, 898 records were determined through database searches. No additional records were identified from other sources. Following the removal of duplicates, 850 unique records underwent title and abstract screening through a dual‐reviewer process. Of these, 556 records were excluded based on the eligibility criteria. The remaining 294 full‐text articles were retrieved and assessed independently by two reviewers for eligibility. Following full‐text assessment, 262 articles were excluded for predefined reasons, resulting in the inclusion of 32 studies in the final scoping review.

Disagreements between reviewers were resolved by discussion and, if unsolved, by a third reviewer (Nina Shenoy). Following full‐text assessment, 32 studies were deemed relevant and included in the final scoping review.

Consistent with PRISMA‐ScR guidance, no formal methodological critical appraisal of included studies was undertaken as the purpose of this review was to comprehensively map the available evidence rather than assess the certainty of evidence. Eligibility assessment and methodological appraisal were therefore treated as distinct processes.

The unit of analysis was the individual publication. Primary studies, secondary reviews, and study protocols were charted separately. Systematic reviews were included to map the breadth of the existing evidence, whereas protocols were included to identify ongoing or planned research. Protocols were not interpreted as evidence of intervention effectiveness.

Records were managed in Rayyan software (https://www.rayyan.ai/), where duplicates were removed prior to screening. The full texts of the remaining 294 reports were retrieved and appraised for eligibility. A PRISMA‐ScR flowchart (Figure 1) details the process, with reasons for exclusions being documented.

**Figure 1:**
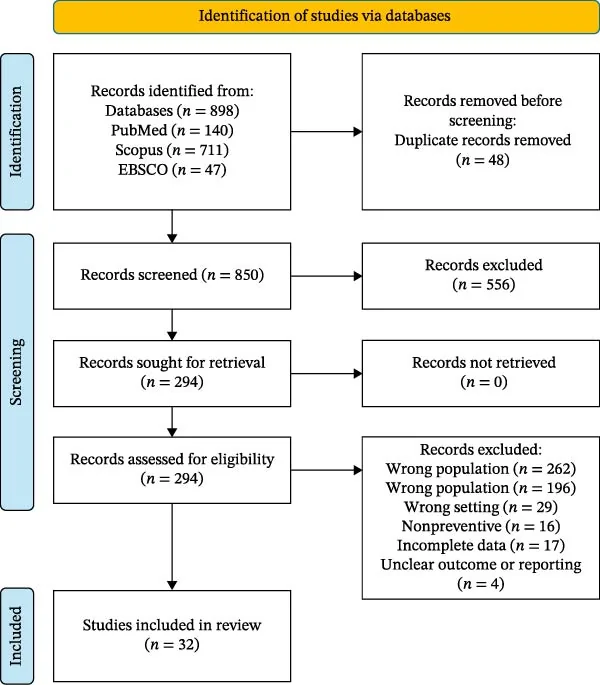
PRISMA‐ScR flow diagram of study selection process for the scoping review on preventive periodontal care for older adults in community settings. Database searches (PubMed *n* = 140, Scopus *n* = 711, and EBSCO *n* = 47) identified 898 records. After duplicate removal (*n* = 48), 850 records underwent title/abstract screening, excluding 556. Full‐text review of 294 articles excluded *n* = 262 (wrong population), yielding 32 studies for thematic synthesis.

### 2.3. Data Extraction and Analysis

Data were extracted independently by two reviewers using a standardized Microsoft Excel data‐charting form. Data extraction used a standardized table with columns: author/year‐citation, study design, setting, population, intervention, deliverer, outcomes, effect sizes, challenges, and solutions, as detailed in Table 2.

**Table 2:** Data extraction sheet: a standardized table with columns—author/year‐citation, study design, setting, population, intervention, deliverer, outcomes, effect sizes, challenges, solutions.

Number	Author, year [citation]	Study design	Setting	Population	Intervention and deliverer		Outcomes	Effect sizes/quantitative results		Challenges/barriers		Solutions/strategies (incl. BCTs, duration, cost)
1	Willumsen et al., 2012 [13]	Cross‐sectional	Nursing home	Elderly residents (*N* = 358 patients)	None (observational assessment + nurse questionnaire)	Nurses	Oral‐hygiene status; factors associated with unacceptable oral hygiene; nurse‐reported barriers	More than 40% of residents had unacceptable oral hygiene. Resistance to assistance was associated with unacceptable oral hygiene (OR 2.5, *p* = 0.018)		Patient resistant behavior most common, followed by lack of time and unpleasantness		Nurse education; organizational changes for time allocation; coping strategies for resistance
2	Czwikla et al., 2021 [9]	RCT	Community (home care)	Older home care recipients (*N* = 527)	Oral health assessment, treatment recommendations, education	Study team (via insurance funds)	OHAT, OHIP, PSI (periodontal screening)	Any periodontal problems were significantly lower in the intervention group than the control group (77.1% vs. 92.0%, *p* = 0.0027). However, periodontitis specifically did not differ significantly between groups (35.4% vs. 44.6%, *p* = 0.1764)		Low participation (5.5%); incomplete delivery/assessment (~37%–57% dropout)		Explore alternative recruitment; more comprehensive interventions (no BCTs/duration/cost specified)
3	Son and Kim, 2024 [19]	Cross‐sectional	Community	Older Koreans ≥65 years (*N* = 3426)	Interdental cleaning device use (floss/brushes)	Self	Periodontal status (CPI ≥3 for periodontitis)	Higher periodontitis risk without floss (AOR = 1.47, 95% CI 1.05–2.1)		Limited research on interdental cleaning in older adults		Interdental cleaning may be considered as part of preventive oral‐hygiene education; effectiveness requires further evaluation
4	Yi Mohammadi et al., 2015 [6]	Systematic review protocol	Nursing home/residential aged care	Older adults with dementia	Professional oral care (mechanical plaque removal, guideline implementation)	Dental hygienists, trained nurses/aides	Plaque scores, general oral health	No outcome data available		Staff prioritization of other care; limited knowledge; resident resistance/violence; neglect cycle		Train‐the‐trainer; hygienist‐led individualized plans; weekly professional care (1 month+); multidisciplinary approach
5	Reisine et al., 2016 [20]	Pilot study	Community (low‐income senior housing)	Older/disabled adults with teeth (*N* = 27)	Bi‐level cognitive‐behavioral (AMI sessions, oral health fairs, resident campaigns)	On‐site team + resident committee	Plaque score (PS), gingival index (GI)	Plaque score decreased from 83% to 58% and gingival index from 1.15 to 0.49 at 3 months		Multiple individual needs/domains (avg 4.5)		Tailored motivational interviewing; community norms via fairs/committee; multilingual materials
6	Van Der Putten et al., 2010 [21]	Cluster RCT	Nursing home/residential care	~24 nursing homes, with ~30 residents per home	Supervised vs. nonsupervised oral health guideline implementation	Nurses/nurse aides	Oral hygiene level	Not reported in abstract (6‐month comparison aimed)		Complicated guideline implementation; resident dependency/neglect		Supervised implementation + daily protocol; process evaluation for barriers/compliance
7	Choong et al., 2026 [1]	Systematic review	Mixed (care homes + community/home)	Dependent older adults ≥60 years	Varied: caregiver training, oral interventions, professional care	Caregivers/health professionals	Plaque index, periodontal status, OHAT, etc.	Findings were highly heterogeneous across interventions and outcome measures		Outcome/measure heterogeneity; limited robust evidence		Develop core outcome set (COS); more robust study designs
8	Johansson et al., 2020 [22]	Feasibility/intervention study	Nursing home	Elderly residents (*N* = 48) + staff	Oral health coaching program (practical staff support)—4 h/week for 3 months	Staff (with coaching support)	DCBS (staff beliefs), oral assessment guide, mucosal‐plaque score	Staff beliefs changed regarding fluoride use, oral‐health support, gum disease and approximal cleaning. Residents’ relatively high oral‐health status remained stable during the study		Program design limitations; general barriers to hygiene in nursing homes		Practical coaching (4 h/week for 3 months); individual‐level staff support
9	Chau et al., 2025 [23]	Pilot study	Community (daycare centers)	Older adults (*N* = 44)	AI mHealth tool (GumAI) for gingivitis detection + personalized instructions	Self (smartphone‐based)	Gingivitis detection (sensitivity/specificity, etc.)	High sensitivity (93%) but moderate specificity (50%) was seen, indicating potential for AI‐assisted gingivitis detection, while further validation in larger and more diverse populations is required		Barriers to routine dental care/access; small pilot sample; diagnostic performance does not establish population‐level effectiveness, feasibility or cost‐effectiveness		AI tool for screening/personalized instructions; high acceptance for community use
10	Chebib et al., 2025 [24]	RCT (single‐blinded)	Nursing home/institutionalized	Institutionalized older adults, *N* = 46	Educational intervention with personalized oral health instruction form (POIF)	Research team/educators	Oral health indices (likely including plaque, etc.)	The intervention group demonstrated significantly better plaque control than the control group after 3 weeks (*p* = 0.023). Denture cleanliness also improved significantly following the intervention. GOHAI scores increased significantly within the intervention group (*p* = 0.006), although the between‐group difference was not statistically significant (*p* = 0.320)		Poor oral hygiene in institutionalized settings		Personalized written instructions as educational tool (BCT: provide instruction); no duration/cost specified
11	Aurlene et al., 2025 [3]	Systematic review	Residential aged care facilities (RACF)	Older people in RACFs	Oral health promotion interventions (varied)	Varied (staff, professionals)	Oral health status measures (general; periodontal not primary)	The review identified substantial heterogeneity in interventions and behavior‐change techniques. Common techniques included instruction and demonstration		Poor oral health in RACFs; evidence for sustained clinical benefit was limited		Analysis of behavior change techniques (BCTs) used; recommend standardized approaches (no specific duration/cost)
12	Tada et al., 2025 [14]	Qualitative descriptive (semi‐structured interviews, thematic analysis)	Long‐term care settings (nursing homes + home‐based medical care)	Medical practitioners in LTC (*n* = 13)	None	Medical practitioners (limited dental integration)	No periodontal measures; focus on awareness of issues (e.g., poor hygiene, decay)	All 13 participants identified effects of oral health on nutrition, while 9/13 discussed aspiration pneumonia		Cultural misconception (aging inevitability); fragmentation of care; mobility/cost/access issues; knowledge gaps in medical training		Education for eldercare community/MPs; domiciliary/mobile dental services; networking/consultative corridors; formal geriatric oral health training (no BCTs/duration/cost specified)
13	Barbe et al., 2025 [25]	RCT	Nursing home	Nursing home residents with periodontitis (*N* not in abstract)	Shortened outreach periodontal therapy (3 months)	Outreach dental team	Periodontal clinical parameters (likely PPD, BOP, etc.)	Between‐group difference in bleeding on probing was significant at 3 months (*p* = 0.041; Cohen’s *d* approximately −0.70, 95% CI −1.40 to 0.00). The PI difference was not statistically significant (*p* = 0.09)		Periodontitis in residents; access barriers		Shortened outreach therapy protocol (3 months duration); mobile/on‐site approach to improve feasibility
14	Azami‐Aghdash et al., 2024 [26]	Systematic review and meta‐analysis	Varied (older people settings)	Older people	Professional oral health care	Professionals (dental/nursing)	Oral health parameters (including periodontal)	Significant improvements in oral health with professional care (meta‐analysis results support positive impact)		Decline in oral health; limited professional care; heterogeneity of intervention types, settings and outcome measures		Increased frequency of professional oral health care; multidimensional maintenance (no specific BCTs/cost)
15	Werbrouck et al., 2022 [7]	Health‐economic evaluation (Markov model)	Nursing home/older patients	Older patients in nursing homes	Oral care interventions (general)	On‐site dental professionals (dentists/hygienists) providing oral care within the facility	Different professional oral‐care strategies	The model estimated the cost per additional healthy oral year for the different strategies as approximately €7944, €1576, and €1132. The likelihood that these strategies were both more effective and less costly than usual care was less than 3%		Findings are model‐based and depend on assumptions and input data, they do not constitute direct clinical evidence of pneumonia reduction		Regular oral care provision; economic evaluation of oral‐care delivery is needed
16	Chen et al., 2020 [2]	Systematic review	Residential aged care facilities	Older people >65 in aged care	Health workforce models for oral health	Varied workforce (e.g., hygienists, nurses)	Oral health needs/outcomes via oral‐health workforce models	The review identified several workforce models involving dental professionals, nurses and integrated approaches. Evidence of short‐term clinical effectiveness was reported in some studies, but the evidence base was heterogeneous		Increasing unmet oral health needs; workforce limitations		Evaluation of models (e.g., integrated workforce); recommendations for addressing gaps (no duration/cost)
17	Barbe et al., 2020 [12]	Intervention study	Nursing home	40 nursing‐home residents; 25 (62.5%) had dementia. Mean age 82 ± 10 years	Regular professional toothbrushing	Dental nurse/professional	Oral health (plaque/hygiene), QoL, nutritional/cognitive status	After 3 months, the Volpe–Manhold Index (VMI), which measures the amount of dental plaque accumulated along the gingival margin, improved significantly (*p* < 0.001). Plaque levels did not change significantly (*p* = 0.239), and gingivitis showed no significant overall improvement (*p* = 0.051). Nutritional status improved in residents with dementia (*p* = 0.012), and oral‐health‐related quality of life also improved (*p* = 0.031)		Poor oral hygiene in residents		Regular professional brushing (efficient method); specialized brush (no duration specified)
18	Manchery et al., 2020 [27]	Systematic review	Nursing home/community	Older adults with dementia and their caregivers	Oral hygiene intervention	Carers (family caregivers and professional nursing staff) after receiving structured oral health education	Oral health parameters, bite force	Four studies were included. All four reported significant improvement in at least one oral‐health measure among older adults with dementia following carer oral‐health education		Evidence was limited due to the small number of studies, and other relevant outcomes were not adequately reported		Intervention adapted to dementia level (no specifics)
19	Samson et al., 2009 [28]	Quasi‐experimental (pretest/posttest, nonrandomized)	Nursing home (Norway)	*N* = 88 residents (both time points); 74F/14M at baseline, 69F/19M at 6‐year follow‐up; elderly nursing home residents	Multicomponent oral healthcare program: (1) staff training (4‐h course), (2) picture‐based oral‐care cards, (3) adequate equipment provision (electric toothbrushes, interdental brushes), (4) oral‐care contact persons per ward, (5) regular MPS screening (6–18 month intervals)	Nursing staff (trained nurses as oral‐care contacts); dental hygienists performed screening; dentists/dental hygienists provided training	Mucosal‐plaque score (MPS); plaque score (PS); mucosal score (MS)	MPS acceptable (≤4): 36% baseline → 70% at 6‐year follow‐up (*p* < 0.001); significant improvement at 3 months maintained at 6 years; kappa = 0.61 for inter‐rater reliability		30% did not achieve acceptable scores due to: (1) very ill/dying (9%), (2) aggressive dementia (5%), (3) mentally alert refusing help (16%); staff knowledge gaps; lack of routines; low priority in busy schedules; ethical concerns about assisting mentally alert residents		Picture‐based procedure cards (6 types for different needs); electric toothbrushes (more effective, less intimate); oral‐care contact system; regular feedback on MPS results to ward administration; systematic protocol; equipment provision; 4‐h training combining theory/practice
20	De Visschere et al., 2011 [11]	Randomized controlled trial (cluster randomization by nursing home)	14 nursing homes in Flanders, Belgium	*N* = 1393 residents total over 5 years (671 control/control, 511 control/intervention, 211 intervention/intervention); 75.9% female; mean age 84.79 ± 7.87 years; 50% highly dependent (Katz scale B/C)	Oral hygiene protocol: (1) introduction session with director, (2) appointed oral health coordinators (registered nurses), (3) half‐day training (train‐the‐trainer), (4) oral assessment of new arrivals, (5) individualized oral hygiene plans	Oral health coordinators (trained registered nurses); all caregivers involved in daily care implementation	Denture plaque (Augsburger and Elahi); dental plaque (Silness and Löe)	Unadjusted: denture plaque lowest at 2 years (CrCh:1.90, CrIh:1.78, IrIh:1.57), faded by year 5 (2.33, 2.04, 2.05); dental plaque reduction baseline to year 5: 0.39(CrCh), 0.16(CrIh), 0.53(IrIh). Adjusted mixed model: intervention NOT significant predictor; denture plaque: capacity (*p* = 0.02), toothpaste presence (*p* = 0.01); dental plaque: dependency (*p* = 0.01), mouth rinse presence (*p* = 0.01)		High staff turnover (caregivers moved between wards/groups); compliance levels uncontrolled; organizational problems (4/14 homes had implementation issues); novelty/Hawthorne effect (initial benefit fading); nursing home characteristics unexplored; resident dependency level (higher dependency = poorer hygiene); lack of caregiver knowledge/skills/experience		Train‐the‐trainer model; individualized oral hygiene plans; oral health coordinators appointed per ward; oral assessment forms for new arrivals; intervention gradually applied to new residents (workload management); 5‐year duration shows need for sustained support
21	Dounis et al., 2012 [5]	Cross‐sectional survey	11 assisted living facilities, Southern Nevada, USA	*N* = 70 (42 M, 28 F); mean age 75.78±SD years; range 34–99 years; 41 had college education; 29 had dental insurance	No intervention—survey‐based assessment only	N/A—descriptive study	Self‐reported oral health status, access to care, oral health problems (survey)	64% rated oral hygiene as fair, 5% as poor, 0% excellent/good; 33% complained of current oral pain; 15.1% toothache, 27.3% gum soreness, 16.3% gum bleeding; 50.4% chewing limitations; 28% halitosis; 59.4% of denture wearers complained about dentures		Facilities did not provide oral health care (58.6%); 96% had not seen dentist since becoming homebound (mean 3.2 ± 2.58 years in program); 61% last dental visit >3 years ago (mean 11.3 ± 8.25 years); difficult access to care (64% reported difficulty); lack of transportation (38% no facility transport); lack of on‐site dental services		93.5% interested in home‐based dental care; specific interests: 81.3% check‐ups every 6 months, 57.7% cleanings, 34.1% help with daily brushing, 38.2% oral hygiene instruction; need for on‐site dental clinics in facilities; integrated health care delivery; workforce training in geriatrics
22	Tashiro et al., 2012 [29]	Quasi‐experimental (within‐subjects, crossover design)	Nursing home, Japan	*N* = 12 dependent elderly; 3 M, 9F; mean age 80.2 ± 6.4 years; ≥10 remaining teeth, no dentures, difficulty with self‐brushing; nursing care levels 3–5	Three independent 5‐day interventions (3‐week intervals): (1) toothbrushing alone (5 min), (2) tongue cleaning with sponge brush (10 wipes per region), (3) wiping oral mucosa with 0.0002% chlorhexidine gluconate	Dentist (20 years experience) and 3 dental hygienists (4–10 years experience)	PlI (Silness and Löe); GI (Silness and Löe); CPI; tongue coat score (Miyazaki); Oral malodor (organoleptic 0–5); saliva wetness; bacterial counts (*P. gingivalis*, *S. mutans*, *Lactobacillus*, streptococci); opportunistic infection pathogens (pharynx)		Toothbrushing: PlI↓ (*p* < 0.01), GI↓ (*p* < 0.01), malodor↓ (*p* < 0.01); CPI, bacteria, saliva wetness NS. Tongue cleaning: tongue coat score↓ (*p* < 0.05), malodor↓ (*p* < 0.01); other measures NS. Chlorhexidine: opportunistic pathogens↓ (*p* < 0.05), malodor↓ (*p* < 0.05); PlI, GI, tongue coat NS	Short‐term (5‐day) intervention insufficient to affect subgingival bacteria, periodontal pockets, caries‐related bacteria; difficult to improve periodontitis by toothbrushing alone without periodontal treatment; some residents with dementia had difficulty accepting electric toothbrushes (noise/vibrations); caregivers lack knowledge/skills/experience for natural dentition care (can clean dentures but not teeth)		Each method targets different outcomes: toothbrushing for plaque/gingivitis; tongue cleaning for coat/malodor; chlorhexidine for opportunistic pathogens; chlorhexidine indicated when pathogens detected; sponge brush effective for tongue/mucosa; short‐term improvement in plaque/gingival outcomes was observed with toothbrushing; need combined approaches based on oral assessment
23	Gluzman et al., 2013 [8]	Cross‐sectional descriptive study	Home‐based (Mount Sinai Visiting Doctors Program, NYC)	*N* = 125 homebound elderly; 80% female, 20% male; mean age 81.42 ± 12.29 years (range 50–100); mean 3.2 ± 2.58 years in program; 28.8% bedridden, 12% wheelchair‐bound	None—comprehensive oral health assessment in subjects’ homes	NYU College of Dentistry research team: 1 trained dental examiner, recorder, assistant; portable dental chair used	DMFT/DMFS; dental status; periodontal status (visual—no probing); soft tissue examination; denture status/hygiene; DUN Questionnaire (10 items); GOHAI (12 items)	76% dentate (mean 14.3 ± 8.0 teeth); 78.9% had ≥1 decayed tooth; DMFT = 25.4 ± 5.43, DMFS = 109.4 ± 28.82; D/D+F ratio = 32.8% (unmet needs); 40% needed restorations, 45.6% needed extractions; 91.6% had ≥1 mobile tooth; 75.8%—gingival inflammation; 71.4%—spontaneous bleeding potential; 30%—teeth hygiene poor (both arches); 33.6% current oral pain; 92% needed some dental care (only 3.2% high urgency, 88.8% moderate urgency)			96% had not seen dentist since becoming homebound; 61% last dental visit >3 years ago (mean 11.3 ± 8.25 years for those who recalled); 33% current pain/discomfort; 50.4% chewing limitations; fragile health preventing invasive examination (no periodontal probing); lack of dental benefits postretirement; substantial logistical barriers to access; no federal mandate for homebound (unlike nursing homes); fragmentation of health care delivery	93.5% interested in home‐based dental care; specific interests: check‐ups (81.3%), cleanings (57.7%), daily brushing help (34.1%), hygiene instruction (38.2%), new dentures (72.2% of denture wearers); portable dental equipment used; need for evidence‐based data to advocate for government/insurance reimbursement; curriculum reform for geriatric dental competence
24	Saarela et al., 2013 [4]	Cross‐sectional survey	Assisted living facilities, Helsinki and Espoo, Finland	*N* = 1447 residents; 79% female; mean age 83 ± 7.5 years; 54.4% primary school education or less; mean 33.7–40.2 months in facility; 84% required ADL assistance; 38% moderate‐severe memory impairment (CDR≥2)	None—assessment of tooth brushing habits and associated factors	Ward nurses (familiar with residents, thoroughly trained) conducted assessments via structured questionnaires	Frequency of teeth/denture cleaning (daily yes/no); dentition type; oral health problems (chewing, swallowing, pain, dry mouth); MNA (nutritional status); CDR (dependency, cognition); Charlson comorbidity index; subjective health; use of dental services	17% (*N* = 252) did not clean teeth/dentures daily; among dentate: 15% did not brush daily; among denture wearers: 11% did not clean daily; only 19% of totally edentulous had daily mouth washing; males worse (27% vs. 15% females, *p* < 0.001); less educated worse (61.6% vs. 54.4%, *p* = 0.052); longer facility stay (40.2 vs. 33.7 months, *p* = 0.003); CDR≥2 memory: 55.4% vs. 34% (*p* < 0.001); ADL dependent: 95% vs. 82.1% (*p* < 0.001)		Dependency in ADLs (*p* < 0.001); cognitive decline/dementia (60% diagnosed with dementia, CDR≥2 associated with less brushing); impaired eyesight (*p* < 0.001); staff attitudes (oral care low priority vs. medical care); shortage of knowledge/training; care‐resistant/uncooperative behavior; lack of equipment/time; malnutrition associated (22% malnourished in nonbrushers vs. 11% in brushers, *p* < 0.001); oral health problems: chewing (39.7% vs. 17.2%, *p* < 0.001), dry mouth (22.7% vs. 15.5%, *p* = 0.011), pain (10.3% vs. 6.4%, *p* = 0.025), swallowing (19% vs. 10.4%, *p* < 0.001); poor subjective health (31.8% vs. 23.5%, *p* = 0.017); less dental service use (37.4% >3 years vs. 26.3%, *p* < 0.001)		Educate nursing staff on significance of oral health; easier to clean complete dentures than natural teeth (89% cleaned dentures vs. 83% brushed teeth); need for daily oral health care as part of nursing care; careful instruction for independent residents while still able; support needed for those unable to perform independently; address practical barriers (equipment, time); manage care‐resistant behavior; recognize oral hygiene as indicator of overall health status
25	Zenthöfer et al., 2013 [30]	RCT	Nursing home	Aged >75 *N* = 106	Staff training + OHI + monthly supervision	Nursing staff	Plaque index, BOP, gingival index	After 12 weeks, plaque, gingival bleeding, and denture hygiene were significantly better in the intervention groups than in the control group (*p* < 0.023). However, these improvements were not maintained at 3 years (*p* < 0.001		Staff aversion to mouth care, time constraints, knowledge gaps (9% found unpleasant)		Staff education + organizational time strategies + regular supervision + positive reinforcement
26	Komulainen et al., 2013 [10]	RCT	Community‐dwelling (home visits)	Aged >75 *N* = 420	OHI + nutrition counseling + repeated education	Community health workers + hygienist	PI, BOP, gingival bleeding, PPD	PI decreased by ~27% and BOP by ~31% at 2 years (*p* < 0.01)		Low OHI adherence, patient resistance, caregiver workload		Repeated education,behavioral counseling, nutrition advice, periodic reinforcement
27	van der Putten et al., 2015 [31]	Cluster RCT	Long‐term care homes (LTC)	Mean age >85 *N* = 342	Supervised OGOLI guideline + staff education + train‐the‐trainer	Nursing staff + WOO (ward oral health organizer)	Dental and denture plaque (Silness–Löe, methylene blue)	Denture plaque ↓ −0.30 ± 0.08 (*p* = 0.007) (significant); dental plaque not significant in multilevel analysis		Nonsupervised implementation less effective, staff resistance, cognitive impairment (75% cognitively impaired); challenges in implementation		Supervised OGOLI implementation + WOO leadership + 6‐weekly monitoring visits + education materials + train‐the‐trainer
28	Lavigne et al., 2017 [32]	Single‐blind RCT	Nursing home (Canada)	Mean age 85.5 *N* = 54	Power toothbrush (twice daily) + mandatory staff training + documentation	Nursing home caregivers	Plaque index (TQHPI), modified gingival index, papillary bleeding index	PI ↓ −0.78, MGI ↓ −0.48, PBI ↓ −0.43 (all *p* < 0.0001) there were no significant differences between the intervention and usual‐care groups		Caregiver aversion, fear of being bitten, aggressive behavior, dementia complicates care (77% had dementia, 72% edentulous in groups)		Mandatory staff training + hands‐on practice with typodont + power toothbrush (improved adherence) + daily brushing documentation + proxy consent protocols
29	Munzenmayer et al., 2019 [33]	Cost‐effectiveness analysis	Aged care facilities (Australia)	Aged >65, 47,624 residential aged‐care places in Victoria, Australia	Professional oral care delivery in residential settings	Dental professionals (mobile/on‐site)	Cost‐effectiveness, measured mainly as cost per pneumonia case averted	Improved oral health; cost‐effective intervention model demonstrated. All four professional oral‐care strategies were estimated to be more effective and less costly than usual care. The most cost‐saving strategy saved about AU$896 per resident, while the most effective strategy was estimated to prevent 6779 cases of pneumonia		Inadequate staff awareness of OHI importance, workload constraints, lack of education, access to dental professionals		Oral hygiene by trained nursing aides twice daily, professional oral‐health care (POHC) once weekly plus oral care by trained nursing aides twice daily on other days, POHC once weekly with current oral‐care provision on other days, and POHC once every 2 weeks with current oral‐care provision on other days
30	Barbe et al., 2019 [34]	Cross‐sectional	Nursing home (Germany)	Varied ages/*N* = 120	Professional dental cleaning (PDC) by dental nurse + feasibility pathway	Dental nurse (3‐year trained)	Plaque index, periodontal status (CPITN)	Time per session: 37 ± 11 min; cost: €14.98/$17.07/session regardless of cognition/mobility status (*p* = 0.803 dementia, *p* = 0.396 mobility)		Staff workload, insufficient education on OHI, dementia, immobility, low prioritization of oral health, resource constraints		PDC as foundational step, clear cost‐effective action pathway, staff training, combined with long‐term strategies (ultrasonic baths, MOUTh programs, Gerodent)
31	McConnell et al., 2018 [35]	Pilot pre–post study (quasi‐experimental; no control group)	Community Living Center—LTC setting	Veterans in LTC with dementia; inclusion: ≥65 years, long‐stay, cognitive impairment; 62 screened, 17 eligible (27%), 11 enrolled; mean age 79 years; all male; 7 dentate	Dementia‐specific oral health program (DOHP): multicomponent oral hygiene program (twice‐daily personalized oral care) including dementia‐specific oral care protocols, personalized oral care plans, unit‐stocked oral hygiene kits, and structured monitoring over 6 months	Existing direct care nursing staff on the CLC (RNs, LPNs, CNAs) trained via multimodal education; “oral care champions” on each unit provided coaching and liaison with the implementation team	Plaque index (PI) and gingival index (GI), measured at baseline, 3 months, and 6 months by a geriatric dentist	PI and GI both decreased over the 6‐month period, indicating reduced plaque and gingivitis; progressive improvement from baseline to 3 and 6 months in the 5 dentate residents who completed all examinations		Multilevel challenges: patient‐level (dementia, resistance to care, agitation/aggression, stroke‐related motor and sensory deficits, depression reducing self‐care); staff‐level (lack of oral care knowledge, lack of dementia‐management skills, no consistent routines for oral care); systems‐level (inadequate staffing, lack of access to toothbrushes/interdental brushes/rinses, no requirement or template for oral care documentation, no personalized oral care plans)		Clear expectations that oral care is a routine ADL; creation of EHR templates for standardized oral assessment, individualized oral care plans, and mandatory daily oral‐care documentation; mandatory multimodal staff training (online + classroom + hands‐on, including role‐play and dementia communication skills); designation and training of unit “oral care champions” for point‐of‐care coaching; weekly interprofessional oral care rounds for bedside coaching, feedback, and problem‐solving; unit‐level stocking of oral care supplies as routine items instead of special order
32	Baumgartner et al., 2015 [36]	Review (narrative/expert discussion paper)	Mixed (community/home care + long‐term care facilities for dependent elderly)	Elderly adults dependent on care	None	Staff/caregivers key (dental hygienists, dentists, nursing staff, and family caregivers in home or institutional settings)	Periodontal measures (BOP/PI/PPD)	Increased incidence and severity of periodontal disease (and caries) in dependent elderly compared with independent older adults		High dependency on care, poor oral hygiene, lack of standardized care concepts, increased caries and periodontal severity, fragmented dental services, and insufficient involvement of dental professionals		Define and implement clear care standards for oral‐health related dentistry; involve dental hygienists/dentists early; improve daily hygiene routines through caregiver training

## 3. Results

### 3.1. Descriptive Summary of Studies

The 32 included articles provided a diverse body of evidence on preventive periodontal care for older adults, with a focus on community and mixed settings. Study designs were varied and included primary studies (cross‐sectional surveys, randomized and quasi‐randomized controlled trials, pilot/feasibility studies, and qualitative studies), secondary evidence (systematic and narrative reviews), and study protocols. These evidence types were considered separately during data synthesis to minimize overlap between primary studies and secondary evidence. Settings predominantly represented nursing homes (*n* = 15), followed by community settings (*n* = 10) and mixed community‐residential settings (*n* = 7). Populations targeted older adults aged ≥60 years (mean ages ranging from 70–85), with sample sizes from small pilots (*N* = 12–48) to large surveys (*N* = 527–3426). Many participants were frail or dependent, often with comorbidities like dementia, mobility issues, or polypharmacy. Figure 2 describes the study settings and study design distribution across the 32 articles.

**Figure 2:**
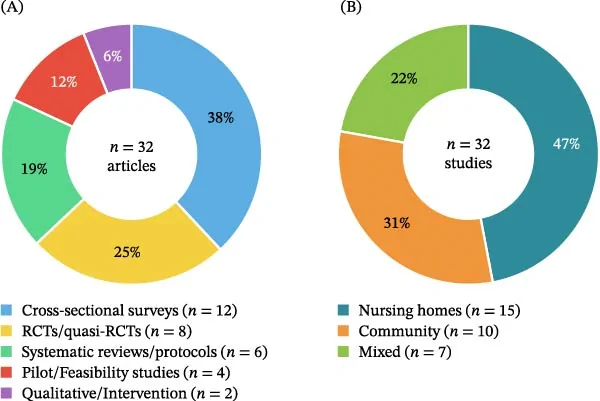
Distribution of study characteristics across 32 included articles in the scoping review on preventive periodontal care for older adults. (A) Study designs: cross‐sectional surveys (*n* = 12, 38%), RCTs/quasi‐RCTs (*n* = 8, 25%), systematic reviews/protocols (*n* = 6, 19%), pilot/feasibility studies (*n* = 4, 13%), qualitative/intervention studies (*n* = 2, 6%). (B) Study settings: nursing homes (*n* = 15, 47%), mixed settings (*n* = 7, 22%), community settings (*n* = 10, 31%). Community settings include assisted living, home‐based care, and daycare centers.

Key periodontal outcomes measured included PI, GI, BOP, PPD, community periodontal index (CPI), mucosal‐plaque score (MPS), tongue coat score, and oral malodor. Effect sizes were generally modest and short‐term, with interventions reducing plaque and gingival indices (e.g., *p*  < 0.05 to *p*  < 0.01), though long‐term sustainability was inconsistent. For instance, short‐term daily interventions (5 days) showed significant reductions in PI and GI (*p*  < 0.01), while longer protocols (3 months to 6 years) improved MPS acceptability from 36% to 70% but left 30% unsatisfactory due to resident factors.

Primary studies informed the synthesis of reported barriers and intervention outcomes, whereas systematic reviews were used to contextualize the findings. Study protocols were mapped as ongoing research and were not interpreted as evidence of intervention effectiveness.

Thematic synthesis revealed two primary domains: challenges and solutions, with subthemes drawn from cross‐study patterns.

### 3.2. Challenges

Challenges were identified at the resident, caregiver, institutional, and systemic levels, often intersecting to create a “downward spiral” of oral health decline [4]. These barriers were prevalent in the majority of articles, with a focus on dependency and access issues in community‐like settings (e.g., assisted living and HBC).

• Resident‐level barriers: cognitive and functional decline among older adults was one of the most consistently reported barriers across the included primary studies, including dementia‐related resistance, motor skill loss, and low self‐perception of oral health. For instance, Saarela et al. [4] note that cognitive impairments (measured by decreased Standardized Mini‐Mental State Examination, i.e. SMMSE scores) correlated with fewer natural teeth and reduced masticatory ability, leading to a spiral of neglect, nutritional deficiencies, and accelerated decline. Willumsen et al. [13] reported “care‐resistant” behaviors (e.g., biting and screaming) in nearly 97% of dementia patients, making daily care difficult. Dounis et al. [5] highlighted self‐rated poor hygiene and irregular dental visits, with residents not prioritizing impairment until function was affected. In homebound contexts, Gluzman et al. [8] described 33% people experiencing oral pain and 92% people with unmet needs due to functional impairment, considering systemic risks like malnutrition.
• Caregiver‐level barriers: knowledge gaps, lack of confidence, and de‐prioritization of oral care were consistently reported across multiple studies. Saarela et al. [4] explained that caregivers view oral hygiene as “grooming” rather than infection control, leading to “missed nursing care”. Tada et al. [14] identified that cultural misconceptions (e.g., tooth loss as an inevitable part of aging) among medical practitioners result in poor care. De Visschere et al. [11] note that nursing staff often find oral care “unpleasant,” prioritizing other tasks due to limited skills.
• Institutional and systemic barriers: workload, inadequate staffing, lack of standardized protocols, and limited access to professional dental care were recurrent barriers reported across studies conducted in nursing homes, assisted living facilities, and home care settings. De Visschere et al. [11] highlighted that high turnover, insufficient manpower, and no structured protocols lead to unsatisfactory plaque levels even after 5 years. Gluzman et al. [8] pointed to logistical barriers (96% had no access to a dentist since being homebound, mean duration of 3.2 years) and absence of mandates in homebound older adults, unlike nursing homes. Dounis et al. [5] reported structural/financial obstacles (64% find access difficult and no in‐house services).

Overall, the included primary studies suggest that these resident‐, caregiver‐, and institutional‐level barriers may contribute to poor oral hygiene, increased plaque accumulation, and reduced access to preventive periodontal care. Several studies also discussed potential implications for systemic health; however, these associations should be interpreted with caution, as the frequency with which barriers were reported across publications does not necessarily reflect their prevalence among older adults [11, 13].

### 3.3. Solutions

#### 3.3.1. Education and BCTs

• The “Train‐the‐Trainer” model: this approach, where a professional (e.g., a dental hygienist) trains a “ward oral health organizer,” has proven effective in increasing the consistency of daily care [6]. Manchery et al. [27] show that carer education for short‐term plaque reductions in dementia patients is effective.
• Motivation‐based programs: long‐term studies show that combining staff motivation with practical tools can yield significant results. Samson et al. [28] described a program with staff motivation, procedure cards, and equipment, improving MPSs from 36% to 70% acceptable over 6 years. Reisine et al. [20] used motivational interviewing and campaigns for GI reductions (1.15–0.49 at 3 months).
• Person‐centered support: programs using person‐centered approaches provided caregivers with specific techniques to manage “care‐resistant” behaviors in residents with dementia, reframing oral care as essential hygiene control rather than a mere grooming task [20].

#### 3.3.2. Standardized Protocols and Tools

Consistency in care is addressed through the implementation of validated assessment tools and innovative technology.

• Standardized assessment: [9].
• Digital and AI innovations: a pilot validation study reported a sensitivity of 93% for detecting gingivitis using an AI‐supported smartphone application. Although promising, further studies are required to establish its clinical effectiveness, feasibility, specificity, acceptability, and cost‐effectiveness before widespread implementation [23].
• Visual guidance: The use of personalized oral health instruction forms (POIFs), which include visual markers and intraoral photos, was associated with greater reductions in plaque accumulation than verbal instructions alone) [24, 28, 30].

#### 3.3.3. Clinical and Outreach Interventions

Bringing professional care directly to community and residential settings circumvents major access barriers.

• Professional Brushing: regular brushing performed by a dental nurse (e.g., every two to 3 weeks) has been shown to improve the nutritional status and quality of life of residents, even when daily nursing staff care is insufficient. Tashiro et al. [29] compared short‐term methods (5 days): toothbrushing reduced PI/GI (*p*  < 0.01), tongue cleaning reduced coat (*p*  < 0.05), and chlorhexidine decreased infections (*p*  < 0.05). Zenthöfer et al. [30] showed that regular professional brushing enhanced QoL and nutrition. Barbe et al. [25] advocated shortened outreach therapy (3 months) for PPD/BOP improvements.
• Mobile and domiciliary services: expanding outreach through mobile dental teams and shortened on‐site therapy cycles can improve clinical markers like pocket depth and BOP in frail populations [14].

#### 3.3.4. Economic and Interdisciplinary Workforce Models

Sustainable solutions require shifting from fragmented care to integrated medical‐dental models.

• Cost‐effectiveness: [2, 7].
• Interprofessional education (IPE): Several studies proposed integrating oral health training into nursing and medical curricula and identifying oral health champions [2] within the workforce; however, these recommendations require further evaluation before routine implementation [2].

## 4. Discussion

This scoping review identifies major challenges and feasible solutions for preventive periodontal care among older adults in community settings. The 32 included sources comprised different study designs and settings, with 15 studies conducted in nursing homes, 10 in community settings, and 7 involving mixed settings. Thus, the available evidence was weighted toward nursing home and other long‐term residential care environments, while evidence from independently living and homebound older adults was comparatively limited. The findings should therefore be interpreted according to the setting in which the evidence was generated rather than generalized across all community‐dwelling older adults [11, 28]. Recent periodontal literature also highlights the necessity of having effective periodontal and peri‐implant management via appropriate preventive and supporting procedures. Contemporary research shows that the results of the procedure can differ depending on the condition, the type of treatment used, and the period of follow‐up, emphasizing the necessity of having individual and evidence‐based periodontal care [15–17]. Specifically, supportive periodontal care is critical for sustaining the results of treatment by preventing the recurrence of the disease and having a stable condition of the periodontal and peri‐implant tissues [15, 17]. The mentioned aspects become especially important when it comes to older adults, who can have physical problems, impaired cognition, dependence, absence of caregivers, and a lack of professional dental care. Consequently, while there is evidence of the necessity of proper periodontal care, its provision remains challenging in practice. Evidence from assisted living conditions and home care reveals heightened challenges when frail patients depend on family caregivers or rotating nonspecialist staff for daily oral hygiene. These findings emphasize the critical role of periodontal care in preserving masticatory function, preventing systemic infections, and maintaining nutritional status in population comprising older adults in such community settings [4, 8].

Problems with memory, manual dexterity, mobility, and cooperation pose significant challenge. For many older adults, especially those with dementia or high levels of dependency, everyday tasks like remembering to brush or having the manual dexterity to clean teeth properly become extremely difficult. This might lead to strong resistance, like turning away or becoming agitated while receiving care, which can overwhelm caregivers [7, 12]. Most of these findings were observed in dependent and institutionalized populations, which may not be representative of all community‐dwelling older adults.

Limited access to professional dental care is another major issue, especially among homebound older adults. One study found that 92% of participants had unmet oral health needs, largely because going to a dentist was difficult because of transportation barriers. Another study reported that 96% of homebound older adults had not seen a dentist for an average of 3.2 years [15].

Transportation, financial difficulties, and limited availability of dental services were reported as important reasons for poor access [5, 15]. However, these figures describe the populations studied in those individual studies and should not be interpreted as the prevalence of unmet dental needs among all homebound older adults.

There were also frequent reports of barriers posed by caregivers. Nursing staff and care workers may be less knowledgeable or confident in doing oral care, especially for older adults with dementia or care‐resistant behaviors [9, 30]. Some studies reported that oral hygiene was not given as much importance as other components of daily care. These findings point to how caregiver education and practical training might enhance the provision of daily oral care. Available studies differ in their interventions, populations, and follow‐up periods. Therefore, the available evidence does not establish that one educational approach is superior to the others.

The delivery of oral care can also be affected by institutional factors. Several researchers reported workloads of staff, staff turnover, lack of training, and lack of standardized oral care procedures [9, 29]. Because of these issues, oral hygiene might depend on personal staff practices rather than being part of the standard [9, 25]. Such barriers appear particularly relevant in long‐term residential settings, while home care settings may face additional problems related to access to dental professionals.

Several possible solutions were identified. Education and behavior‐change approaches, including train‐the‐trainer programs and person‐centered strategies, were associated with improvements in oral care practices or short‐term oral health outcomes in some studies [16, 18, 31, 35].

Caregivers can learn the importance of oral care and manage the difficulties associated with dementia through these approaches. Nonetheless, the existing evidence is constrained by variations in the intervention design, sample size, context, and follow‐up duration. To understand whether these benefits can be sustained over the long term, additional research is required.

Regular oral care can be supported by structured assessment tools and visual instructions. The Oral Health Assessment Tool (OHAT) can help non‐dental staff identify oral health problems and when referral may be needed [6]. However, it is a general OHAT and not a substitute for a periodontal examination or diagnosis. Like personalized visual instructions have been beneficial in certain studies [28], their use in other community and residential settings requires further examination.

Digital technologies are an emerging area. An AI‐assisted smartphone app has reported a sensitivity of 93% for detecting gingivitis [27], but this result alone is not enough to establish specificity, efficacy, feasibility, acceptability, and cost‐effectiveness.

Interventions aimed at professionals and outreach efforts might also help with improving access to dental care. While interventions, which included professional brushing of teeth, domiciliary care, mobile clinics, and other types of outreach activities, showed some improvement in the oral or periodontal status of residents [10, 20, 21, 24], it should be noted that due to the differences in setting, design of interventions, and follow‐up period, evidence suggests the possible benefits but cannot confirm any long‐term periodontal and systemic effects of the abovementioned interventions among all older people.

Economic evidence was also limited, as there was no information that can prove the cost advantages of on‐site care in comparison with other approaches [8, 22]. The cost‐effectiveness needs further examination. In general, several useful approaches were identified during the examination of existing literature related to preventive periodontal care, such as oral‐care protocol, caregiver education, regular oral health assessment, proper referral pathway, and outreach services. However, the gathered evidence is not solid enough to make certain measures compulsory, such as professional training and using mobile periodontal clinics [12, 25, 28–30, 34].

### 4.1. Evidence Limitations

The reliability and transferability of the evidence are constrained by the heterogeneity of the included studies. Nursing home residents represented a large proportion of the subjects, whereas independent living or homebound older individuals were not well represented. Many studies were cross‐sectional or had small samples and short follow‐up. Qualitative research provided valuable information about experiences and barriers; however, qualitative data is not an indicator of the prevalence of these issues. Similarly, the number of articles reporting a barrier also do not give an idea of its prevalence. Reviews and protocols gave the background, whereas systematic and narrative reviews provided context; however, neither type provides evidence for effectiveness. Furthermore, including secondary reviews along with primary research increases the chance of overlapping data in the evidence base, thus increasing the risk of some results being considered twice.

Other limitations include the restriction to English‐language publications, which may have introduced language bias, and the 2010 publication date limit, which was intentionally applied to exclude older literature that was considered less relevant to current clinical practice, but this may still have resulted in the omission of some earlier evidence.

Future research should concentrate specifically on independent older adults and those who live at home. The well‐designed longitudinal and controlled study should investigate caregiver education, structured oral care, professional support, and outreach service and specify the type of setting, dependency of the participants, intervention, comparison group, duration of follow‐up, and periodontal outcomes. Health economic research is necessary in order to estimate the cost‐effectiveness of different models of care. Research in various geographic and socioeconomic settings will make the evidence more applicable. Future studies could also include inflammation and other systemic outcomes in order to investigate the systemic effect of periodontal preventive care, but the outcomes should not be assumed but proved.

## 5. Conclusion

This scoping review highlights the persistent multilevel barriers to delivering effective preventive periodontal care for older adults in community and mixed residential settings. Issues such as cognitive and physical decline in residents, low prioritization of oral hygiene, gaps in caregiver knowledge and confidence, heavy workloads with less caregiver staff, and poor access to professional dental support often lead to inadequate plaque control. These problems, in turn, increase the risk of malnutrition and aspiration pneumonia and hence cause a noticeable decline in quality of life.

Several practical solutions appear promising, including tailored training programs for caregivers that incorporate BCTs, simple tools like the OHAT, visual aids with intraoral photographs, AI‐assisted screening apps, regular professional outreach brushing, and stronger collaboration between dental and medical teams. However, most of these interventions only produce modest, short‐term improvements in key periodontal outcomes, namely, PI, GI, and BOP.

Overall, the findings highlight the need to develop integrated preventive strategies that work across both community and residential environments. Policymakers should consider making oral health protocols mandatory in community care systems (similar to what is already required in many nursing homes) with regular staff training, mobile and accessible dental services, and better interprofessional support to address the needs of homebound older adults.

Although this review provides useful insights, limitations still exist, especially the heavy reliance on nursing home data and the lack of long‐term studies focused specifically on community settings, especially in low‐resource contexts. Future research should prioritize cost‐effectiveness evaluations, biomarker assessments, and well‐designed trials in real‐world community and rural settings. Strengthening preventive periodontal care in these ways could make a meaningful difference to both oral and general health in our rapidly aging population.

## Author Contributions

**Ankita Chakraborty**: conceptualization, methodology, data curation, formal analysis, investigation, writing – original draft, writing – review and editing. **Deepa Kamath G**: conceptualization, methodology, supervision, validation, writing – review and editing, project administration. **Nina Shenoy**: writing – review and editing, validation. **Anwesha Basu and Aradhya Sinha**: writing – review and editing.

## Funding

This study did not receive any specific funding.

## Disclosure

All authors have read and approved the final version of the manuscript. Deepa Kamath G had full access to all the data in this study and takes complete responsibility for the integrity of the data and the accuracy of the data analysis. This study was presented for the 29th IAPHD NATCON conference (Registration Number 578).

## Ethics Statement

Ethical approval was not required for this scoping review as it involved secondary analysis of previously published data only.

## Consent

The authors have nothing to report.

## Conflicts of Interest

The authors declare no conflicts of interest.

## Data Availability

The authors confirm that the data supporting the findings of this study are available within the article. Preferred Reporting Items for Systematic Reviews and Meta‐Analyses Extension for Scoping Reviews (PRISMA‐ScR) checklist has been provided as a form.
